# Incurability of Chronic Leucorrhea

**Published:** 1871-05

**Authors:** 


INCURABILITY OF CHRONIC LEUCORRHEA.—It may be a useful lesson to some medical writers, who make no failures, or who are afraid to acknowledge them, to learn that so distinguished a practitioner as Scanzoni announces publicly that he has never cured a case of chronic leucorrhea.—*Pacific Medical Journal.*

